# Crystal forms in pharmaceutical applications: olanzapine, a gift to crystal chemistry that keeps on giving

**DOI:** 10.1107/S2052252520012683

**Published:** 2020-10-03

**Authors:** Susan M. Reutzel-Edens, Rajni M. Bhardwaj

**Affiliations:** a Synthetic Molecule Design and Development, Eli Lilly and Company, Indianapolis, IN 46285, USA

**Keywords:** olanzapine, polymorphs, hydrates, crystal forms, pharmaceuticals, crystallization

## Abstract

This contribution reviews the efforts of many scientists around the world to discover and structurally characterize olanzapine crystal forms, clearing up inconsistencies in the scientific and patent literature and highlighting the challenges in identifying new forms amidst 60+ known polymorphs and solvates. Owing to its remarkable solid-state chemistry, olanzapine has emerged over the last three decades as a popular tool compound for developing new experimental and computational methods for enhanced molecular level understanding of solid-state structure, form diversity and crystallization outcomes. This article highlights the role of olanzapine in advancing the fundamental understanding of crystal forms, interactions within crystal structures, and growth units in molecular crystallization, as well as influencing the way in which drugs are developed today.

## Abstract 2

The rich solid-state chemistry of olanzapine uncovered during the development of this blockbuster drug has inspired the development and application of new tools and techniques for understanding molecular assembly during crystallization.

## 1. Introduction   

Every molecule has a story to tell. This is what years of experience in the pharmaceutical industry have taught us. However, with high molecule attrition in drug development, an estimated 1 in 10 000 compounds in discovery pipelines will make it to the market (PhRMA, 2013 ▸); most of their stories will never be told. For the lucky few molecules that do make it, an incredible amount of information on solid-state forms and their properties is likely to be amassed over the many years it takes to transform a molecule into medicine. Knowledge is inevitably accumulated while identifying and quantifying crystalline solid forms, characterizing their structures and properties, developing processes to crystallize them for purification and downstream storage, and formulating them for eventual delivery to the patient. Blockbuster drugs are likely to attract further attention by generic companies seeking early entry in the marketplace by circumventing or invalidating crystal form patents of the innovator. Of course, molecules of commercial significance also make for excellent substrates to test new and emerging experimental and computational methods.

Most drug candidates undergo some form of crystallization screening early in development to identify viable crystal forms, with more comprehensive surveys of the solid form landscape reserved for promising candidates that progress into commercial development. In our experience, well studied compounds are more likely than not to crystallize in multiple forms, polymorphs and hydrates (Cruz-Cabeza *et al.*, 2015 ▸; Stahly, 2007 ▸), although these forms are not always discovered during solid-form screening. As these discoveries are generally made over time, it is rare to find a single source of information on all aspects of the solid forms, especially for blockbuster drugs. Instead, reports on their solid-state chemistry will necessarily reflect what is known at the time, and in some cases, may be intentionally limited to a particular finding or development. This inevitably leads to a proliferation of reports on various aspects of the solid-state chemistry of important drug molecules. Among the many pitfalls of having disparate sources of information are that authors are not always aware of precedent in the literature, not all disclosures are peer-reviewed to help reduce the number of false or misleading claims, some reports may be seemingly or outright conflicting, and over time it becomes increasingly difficult to ‘see the forest for the trees’. Just as individual chapters fall short of telling the whole story, isolated reports of the solid-state chemistry of a drug do not usually reveal how the compound was developed into a solid oral dosage form, what the challenges were, and the role that crystals and crystal chemistry played in navigating the complex process that is drug development.

**Figure 1:**
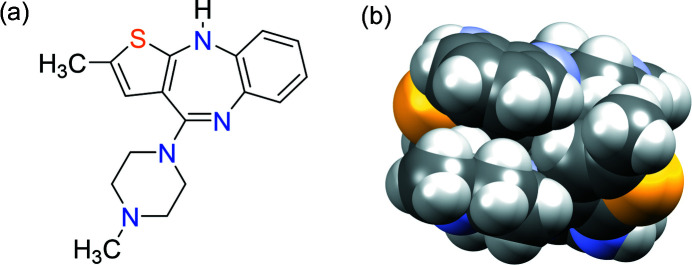
(*a*) Molecular structure of OZPN and (*b*) dispersion-bound head-to-tail OZPN dimer.

In this contribution, we look back at many of the important discoveries and insights into the crystal chemistry of olanzapine {2-methyl-4-(4-methyl-1-piperazinyl)-10*H*-thieno-[2,3-*b*] [1,5]benzodiazepine, OZPN}, an atypical antipsychotic agent originally marketed as Zyprexa for the treatment of bipolar disorder and schizophrenia (Fulton & Goa, 1997 ▸; Cansever & Battal, 2000 ▸), see Fig. 1 ▸(*a*). OZPN is not just known to crystal chemists and crystallographers for having given us the spectacularly polymorphic molecule, ROY, a synthetic precursor characterized by its red, orange and yellow coloured crystals (Yu, 2002 ▸, 2010 ▸; Yu *et al.*, 2005 ▸; Gushurst *et al.*, 2019 ▸; Lévesque *et al.*, 2020 ▸; Tyler *et al.*, 2020 ▸; Nyman *et al.*, 2019 ▸; Li *et al.*, 2020 ▸). The drug compound, having been crystallized in 60+ forms (not including salts and co-crystals) over the course of more than two decades, is famous in its own right. Not only did the complex crystal chemistry of OZPN provide challenges to the development of this blockbuster drug at every turn, but it is also safe to say that with each new study, OZPN continues to challenge our collective understanding of how molecular crystals nucleate and grow. Here, we endeavour to connect published reports and observations made over many years to tell what we hope is a cohesive story of an incredible molecule, a gift to crystal chemistry that keeps on giving.

## 2. Polymorphs, patents, perversity   

Early in its development at Lilly, OZPN revealed itself to be easily and highly crystallizable, able to crystallize by itself, but particularly susceptible to forming solvates with a wide range of solvents (Bhardwaj *et al.*, 2013 ▸). Among the many crystal forms, two anhydrous polymorphs, forms I and II, were identified. Whereas form II was usually obtained by desolvation, form I could be directly crystallized from solution from the relatively few solvents in which OZPN did not form a solvate. Form I was confirmed to be the thermodynamically most stable neat form, and with excellent solid-state properties (thermal stability, hygroscopicity, morphology, *etc*.) and just enough solubility, this form was selected for commercial development and ultimately marketed in Zyprexa.

It should be noted that form I was in fact discovered *after* form II and so the naming convention in the patent literature reflects the order in which the two polymorphic forms were discovered. The discovery of the stable form came at a time when it was fashionable (at least to some) to reserve ‘form I’ for the stable form: a practice that is *not* to be encouraged. As a result, the stable form was designated form I, and the metastable polymorph, which was discovered first, was renamed form II, and this nomenclature was used thereafter at Lilly, including in the Zyprexa regulatory submission. In keeping with the regulatory documentation, we introduced this naming convention to the scientific literature in our 2003 report on the anhydrous and hydrate crystal forms of OZPN (Reutzel-Edens *et al.*, 2003*a* ▸).

**Figure 2:**
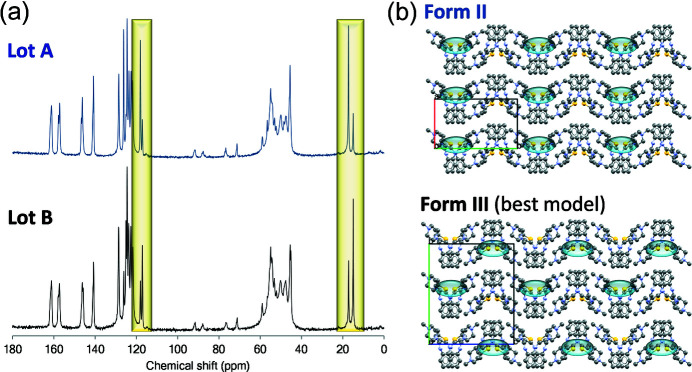
(*a*) Solid-state ^13^C NMR spectra of two OZPN form II/III mixtures (lots A and B). The regions showing different relative peak intensities, the clearest indication of a phase mixture, are highlighted. (*b*) Crystal structure views of form II (CSD refcode: AQOMAU03) and the closest CSP match to form III (structure A162). Outward facing thio­phene S atoms (yellow) are highlighted to emphasize the stacking difference between the experimental form II and computed form III crystal structures.

In the mid 1990s, long after forms I and II were discovered (and patented), we determined that the metastable form (form II) had never been isolated as a phase-pure material. Instead, form II materials were mixtures of two metastable neat polymorphs, hereafter referred to as forms II and III. While any technique capable of distinguishing crystal forms may, in principle, be used to identify them, it is generally recognized that not all techniques are created equal and combinations of analytical tools are sometimes needed (Yu *et al.*, 1998 ▸). In fact, the presence of a form III phase impurity was not obvious from the powder X-ray diffraction patterns, FTIR spectra or DSC curves which had previously been used to identify OZPN crystal forms. It was not until solid-state ^13^C NMR spectroscopy, a relatively new entry into our arsenal of characterization tools, arrived that clear differences were seen between different ‘form II’ materials which could only be ascribed to different amounts of a phase impurity being present (Reutzel-Edens *et al.*, 2003*a* ▸). As shown in Fig. 2 ▸(*a*), the ssNMR spectra of two form II/III lots (A and B) are strikingly similar, but with notably different relative peak intensities at 15–20 and 115–120 p.p.m.

Forms II and III are produced concomitantly, either as a pair or mixed with form I, by desolvation of certain OZPN solvates under mild conditions, neat grinding, sublimation, recrystallization of amorphous OZPN, spray drying and freeze drying. Despite considerable effort over many years and by different groups around the world to crystallize OZPN, the two metastable polymorphs have not, to the best of our knowledge, ever been produced as pure polycrystalline phases. Single crystals of form II have, however, been grown by two different methods, one a failed attempt to cocrystallize OZPN with nicotinic acid (Thakuria & Nangia, 2011 ▸) and the other by sublimation of pure OZPN (Bhardwaj *et al.*, 2013 ▸). On both occasions, the crystal structure of form II was solved, first at room temperature, then at 123 K. Comparison of the simulated form II powder patterns from the single-crystal X-ray diffraction (SCXRD) structures and the experimental powder X-ray diffraction (PXRD) data corroborated the early ssNMR finding of a form III phase impurity present in all samples of the metastable form II.

Unable to grow form III single crystals for structure determination, an attempt was made to identify its structure by two-phase Pawley-type refinement. The lattice parameters from the form II crystal structure and the 50 lowest energy structures from a crystal structure prediction (CSP) study (Section 3), along with the best available form II/III mixed-phase PXRD pattern, were used for the refinement. The final form III structure model, in combination with form II, provided the best statistical fit, but was not a perfect match to the PXRD pattern (the refinement did not account for a few peaks assumed to be form III). However, it appeared to be sufficiently close to identify form III as a layered variation of form II, see Fig. 2 ▸(*b*) (Bhardwaj *et al.*, 2013 ▸). This was important for understanding why desolvation always leads to the concomitant formation of forms II and III.

The commercial success of Zyprexa provided an enormous incentive for the generic drug industry to find new crystal forms of OZPN or ways to get around the known (patented) forms. Along these lines, efforts were directed to producing products with the metastable form that was first to come off patent, to invalidating the stable form patent and to finding altogether different forms that could be used to deliver OZPN to patients. Soon reports of new polymorphic forms of OZPN would surface in the scientific and patent literature, which if true, would raise the total number of known neat OZPN polymorphs to at least six (Wawrzycka-Gorczyca *et al.*, 2007 ▸; Cavallari *et al.*, 2013 ▸). Rest assured, there are not as yet six confirmed non-solvated polymorphs of OZPN. Several claims to new neat forms were presumably cases of mistaken identity, largely based on misinterpretation of PXRD patterns of physical mixtures of known phases. Alas, some publications (Tiwari *et al.*, 2007 ▸), as well as patent claims to a new OZPN form III, overlooked or ignored our 2003 report identifying a third polymorph (III) in mixture with form II, and while the existence of form III was recognized in a 2011 publication reporting a newly discovered form IV (Thakuria & Nangia, 2011 ▸), excellent agreement between the PXRD pattern simulated from the single-crystal structure disclosed in this report and the experimental powder pattern of form II quickly disproved the claim of a new polymorph. Only recently has a fourth OZPN polymorph, form IV, been confirmed (Askin *et al.*, 2019 ▸), and as will be discussed later, its discovery would wait many years for the right heterogeneous nucleation experiment to be conducted.

**Figure 3:**
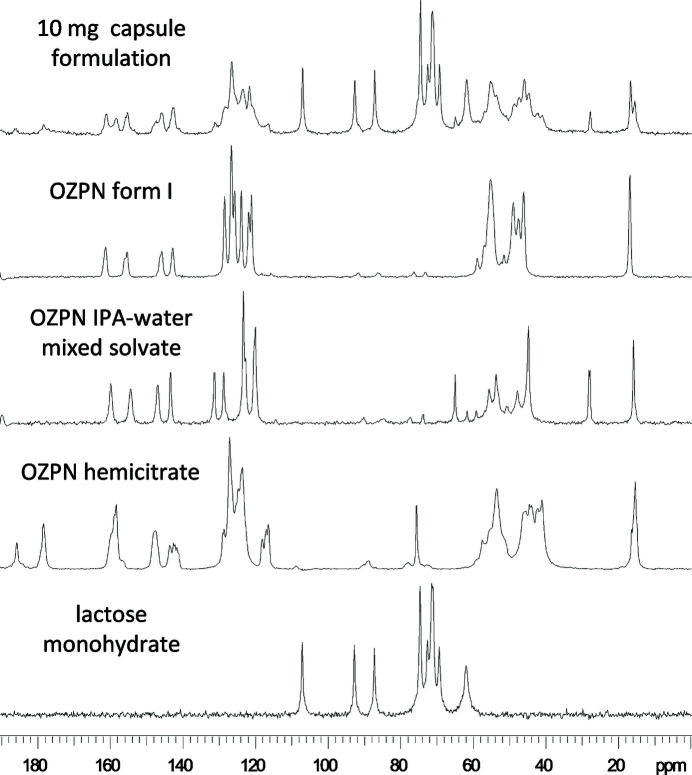
ssNMR spectrum of a generic OZPN 10 mg tablet, along with reference spectra of ^13^C-containing components, including three different crystal forms of the active ingredient alone.

The task of identifying new phases amid the >50 crystal forms that emerged during the solid-form screening and crystallization process development of OZPN was daunting. Laboratory PXRD, thermal analysis and FTIR spectroscopy, which were used in support of all OZPN patent claims to new forms, were clearly not up to the task. Synchrotron PXRD, although sufficiently discriminative (Testa *et al.*, 2019 ▸), was not available at the time. Therefore, with the form III polymorph so clearly revealed for the first time by ssNMR spectroscopy (Fig. 2 ▸), this powerful technique would soon become our workhorse for identifying OZPN crystal forms. In fact, ssNMR spectroscopy featured prominently in our efforts to not only identify new crystal forms throughout the development of Zyprexa, but also demonstrate infringement of patents throughout the product lifecycle. Shown in Fig. 3 ▸ is one of many examples of generic drug products analyzed over the years where ssNMR spectroscopy showed just how difficult controlling the solid form of OZPN during pharmaceutical processing and storage can be. Here, a mixture of at least three different crystal forms of OZPN, including the patented form I, are clearly identified in one tablet formulation.

## 3. Landscapes, leads, learning   

At the time of writing, OZPN has four confirmed neat polymorphs (I–IV), see Table 1 ▸. But are there others to be found, and if so, would any of them be sufficiently stable to pose a risk of a disappearing polymorph event (Bučar *et al.*, 2015 ▸) should they suddenly appear? These questions have concerned the pharmaceutical industry ever since the high-profile Ritonavir crisis in 1998. When the crystallization of Ritonavir form II, a more stable, less bioavailable polymorph, forced Abbott to withdrawal Norvir capsules from the market (Bauer *et al.*, 2001 ▸; Chemburkar *et al.*, 2000 ▸), the pharmaceutical industry rallied to avoid similar crises in the future, pouring millions of dollars into solid-form screening programs. However, expansion of the tools and techniques for solid-form screening, including the development of high-throughput crystallization platforms, did not prevent late-appearing polymorph catastrophes (*cf*. Rotigotine) (Rietveld & Céolin, 2015 ▸; Mortazavi *et al.*, 2019 ▸). This meant that in the search for neat polymorphs (and hydrates), experiments would continue until one could be reasonably confident that at least the stable form had been found. For monomorphic compounds and those that readily crystallize in the stable form, exhaustive solid-form screening is clearly not needed. Even for OZPN, the amount of effort that has produced just four polymorphs, none more stable than form I, has been enormous.

Eager to reduce the experimental footprint of solid-form development by not unnecessarily prolonging solid-form screening, the pharmaceutical industry has now turned to computational chemistry, specifically CSP, to assess the completeness of solid-form screens (Price *et al.*, 2016 ▸; Nyman & Reutzel-Edens, 2018 ▸). CSP is a computational methodology for generating and ranking by energy the different ways that a molecule can pack in three-dimensional crystal structures, starting with a chemical diagram of the molecule. OZPN, having been extensively screened for polymorphs, was an early test for the CSP algorithms being developed for larger, flexible pharmaceutical molecules.

**Figure 4:**
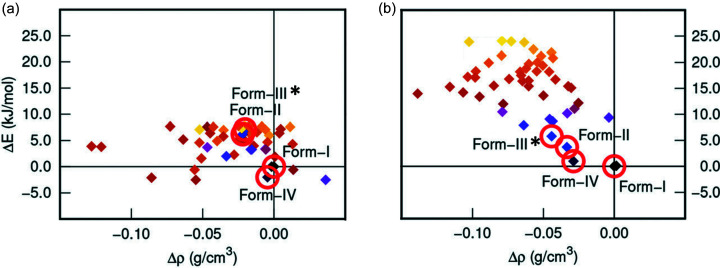
Crystal energy landscape of OZPN (*a*) from the CSP study (Bhardwaj *et al.*, 2013 ▸) and (*b*) after reranking with single-point energy calculations using plane-wave B86bPBE-XDM (PAW). Structures corresponding to experimentally observed forms I, II, IV and the best structure model of form III, are highlighted. Energies and densities are referenced to the most stable polymorph, form I (adapted from LeBlanc & Johnson, 2019 ▸ with permission).

The search for possible OZPN structures, which was limited to two low-energy regions of conformational space, produced dozens of crystal structures as minima within the energy range of the experimentally observed polymorphs, see Fig. 4 ▸(*a*). The CSP study found structures corresponding to the three polymorphs that were known at the time, including exact structure matches to forms I and II, and a close structure match (A162) to form III (hereafter referred to as III*), see Fig. 2 ▸(*b*). These three structures and several predicted, yet unobserved low-energy structures, each featured centrosymmetric dispersion-bound OZPN dimers [Fig. 1 ▸(*b*)], the crystal building blocks seen in all 56 structurally characterized (by SCXRD and PXRD) solvates (Bhardwaj *et al.*, 2013 ▸). It is noteworthy that only dimer-based neat forms have been observed, given that most of the thermodynamically competitive structures on the crystal energy landscape do not contain dimers. With PIXEL calculations (Gavezzotti, 2002 ▸, 2003 ▸ *a*,*b* ▸; Dunitz & Gavezzotti, 2005 ▸) showing dispersion forces to be the strongest of the individual pairwise intermolecular interactions in OZPN crystal structures, it was surmised that OZPN dimerizes in solution at the pre-nucleation stage (Section 5), reducing the concentration of single-molecule building blocks needed for alternate, non-dimer based structures to nucleate and eventually grow.

The appearance of form II and III* structures in the high-energy region of the crystal energy landscape was not unexpected for these desolvate polymorphs and finding the form I structure, which could be crystallized directly from solution, near the bottom of the energy window was reassuring. However, other structures were competitive in energy with form I, including a few calculated to be slightly *more* stable at 0 K. Among these were structures not based on dispersion-bound OZPN dimer assembly. With a growing number of reports of predicted crystal structures having later been found (Braun *et al.*, 2014 ▸, 2016 ▸; Arlin *et al.*, 2011 ▸; Neumann *et al.*, 2015 ▸) and the potential to crystallize a more stable polymorph, many attempts, all unsuccessful, were made to target non-dimer structures of OZPN (Bhardwaj *et al.*, 2013 ▸). Eventually, a fourth polymorph (IV), not based on OZPN dimers, would be discovered using a novel screening approach: heat-induced crystallization from an amorphous polyvinyl­pyrrolidone (PVP) based molecular dispersion (Askin *et al.*, 2019 ▸).

The prediction of more stable OZPN crystal structures prompted further exploration of crystallization conditions which ultimately led to the discovery of form IV. However, any decision to invest resources in additional solid-form screening must consider that the ranking of CSP structures depends strongly on the energy model and usually neglects the effects of temperature. In fact, energy reranking of the 0 K structures of OZPN using a higher accuracy energy model, specifically B86bPBE-XDM (PAW), produced a crystal energy landscape with the four lowest energy candidate structures corresponding to the four known polymorphs, see Fig. 4 ▸(*b*) (LeBlanc & Johnson, 2019 ▸; Luo *et al.*, 2019 ▸). Forms I and IV remain, within the margin of error, comparable in lattice energy and more stable than forms II and III*. A more useful measure of stability is, of course, the Gibbs free energy. Calculation of the free energy can determine which low energy 0 K structures are not free energy minima and help to identify those that are thermodynamically competitive at ambient and process-relevant temperatures. Recently, the relative free energies of OZPN forms I and II were calculated using embedded fragment quantum mechanical methods. Form I was found to be monotropically more stable than form II, in agreement with experiment, with an energy difference of *ca* 4.4 kJ mol^−1^ at 298 K (Luo *et al.*, 2019 ▸).

## 4. Hydrates, hygroscopicity, headaches   

OZPN has been structurally characterized in four different hydrates: three polymorphic dihydrates and a 2.5 hydrate (also named higher hydrate), see Table 1 ▸. The path to finding the crystalline hydrates strangely parallels the discovery of the neat OZPN polymorphs. Dihydrates B and D, like forms I and II, were found early in development. These hydrates crystallize directly from aqueous–organic solutions, although they could just as easily be formed by slurrying the neat polymorphs in water. Dihydrate B is the kinetic hydrate, which appears first in aqueous suspensions, but eventually gives way to the thermodynamically more stable dihydrate D. As with form III, dihydrate E eluded detection until it was first identified by ssNMR spectroscopy, usually in batches mixed with dihydrate B. Fortunately, experimental conditions were identified to reproducibly and selectively produce phase-pure polycrystalline samples of the three dihydrates (Reutzel-Edens *et al.*, 2003*a* ▸).

**Figure 5:**
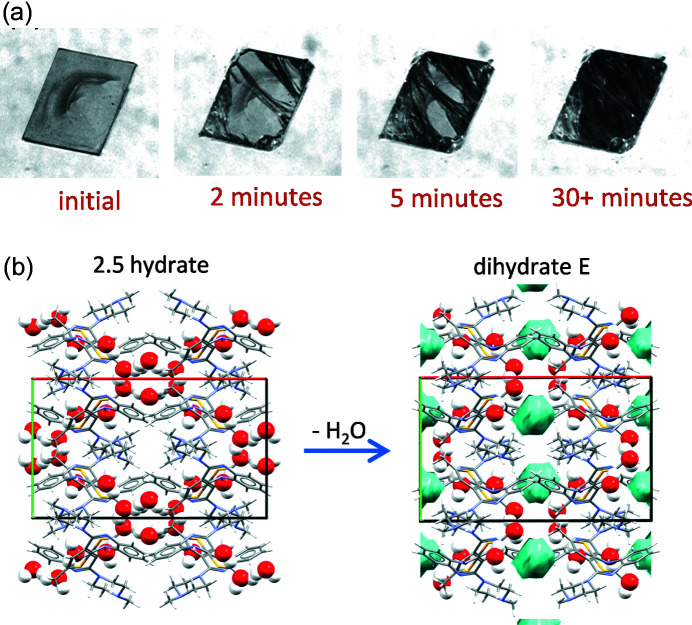
(*a*) Optical micrographs showing rapid conversion (desolvation) of the freshly crystallized 2.5 hydrate to dihydrate E, and (*b*) crystal structure views of the parent 2.5 hydrate [CSD refcode: AQOMEY02] and product dihydrate E. With minimal change to the unit-cell dimensions upon dehydration of the 2.5 hydrate, voids (shown in light blue) are created in dihydrate E, where 0.5 molar equivalents of water were lost below 75% RH.

High-quality single crystals of dihydrates B and D were grown allowing their structures to be solved by SCXRD, and following the discovery of dihydrate E, countless attempts were also made to grow single crystals of this third dihydrate. We were never successful in this endeavour and the reason why our repeated attempts to solve the structure of dihydrate E by SCXRD failed was not immediately obvious, that is, until we carefully examined a freshly grown single crystal harvested from our best dihydrate E preparation under a microscope. As shown in Fig. 5 ▸(*a*), translucent single crystals of very high quality could be grown, but they were highly unstable once removed from the ethyl acetate–toluene–water mother liquor, desolvating within minutes under ambient conditions. It had become clear that dihydrate E was not the form that crystallized directly from solution; instead, dihydrate E was a [partial] desolvation product. To identify the parent OZPN crystal form crystallized from solution, we collected diffraction data on a freshly grown crystal that was immediately cooled to low temperature to prevent loss of solvent. The crystal structure was successfully solved, showing for the first time that OZPN had crystallized from solution in what appeared to be a 2.5 hydrate (Reutzel-Edens *et al.*, 2003 ▸ *a*).

Unable to grow single crystals of dihydrate E, the structure of this dihydrate was eventually obtained by Rietveld refinement of a structure model derived from the isostructural ethanol–water mixed solvate (CSD refcode: WEXQEW) against laboratory PXRD data (Reutzel-Edens *et al.*, 2003 ▸ *a*). Dihydrate E is isostructural to the 2.5 hydrate (Fig. S4 of the supporting information) and so there appears to be minimal disruption to the unit cell with the loss of 0.5 molar equivalents of water from the parent hydrate. However, based on polarized light microscopy, the partial dehydration does not proceed via a single crystal-to-single crystal transformation. Instead, the loss of the most weakly bound water from isolated pockets in the 2.5 hydrate crystal structure [Fig. 5 ▸(*b*)] is sufficiently disruptive to transform the translucent 2.5 hydrate crystals into striated, opaque polycrystalline dihydrate E particles [Fig. 5 ▸(*a*)], retaining the overall shape of the parent hydrate. The dihydrate E experience serves as a useful reminder that crystalline products, once harvested, may not reflect what actually crystallized from solution.

**Figure 6:**
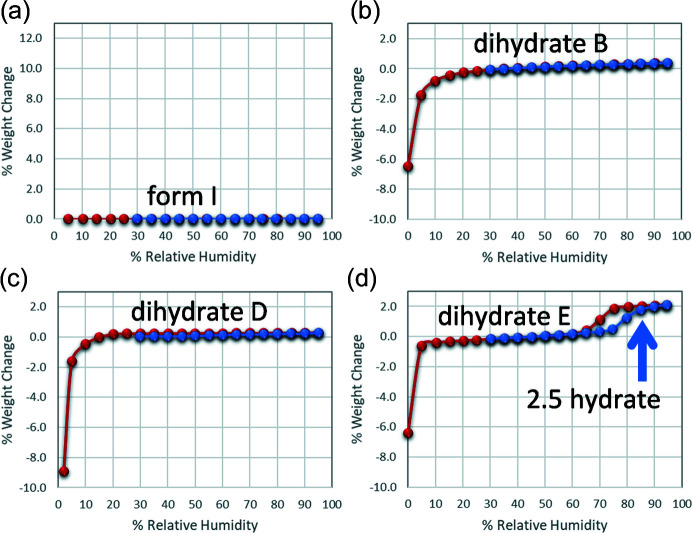
Gravimetric water vapour sorption–desorption isotherms of OZPN (*a*) form I, (*b*) dihydrate B, (*c*) dihydrate D and (*d*) dihydrate E/2.5 hydrate. Water content was measured in 5% RH increments starting at 30% RH, increasing to 95% RH (blue circles), then decreasing to 0% RH (red circles).

Water vapour sorption analysis can be an effective way to examine phase stability as a function of relative humidity (RH) and is often used to screen for crystalline hydrates, desolvates and exchange products, some of which may be inaccessible by solution crystallization. Although dihydrates B and D are known to form in aqueous suspensions, form I showed no signs of hydrate formation in the solid state between 5 and 95% RH during a typical (∼24 hour) gravimetric vapour sorption (GVS) experiment, see Fig. 6 ▸(*a*). By contrast, evidence of form conversions involving changes in water content was observed in the room-temperature GVS isotherms of the OZPN dihydrates, see Figs. 6 ▸(*b*)–6(*d*). All three dihydrates are rendered unstable with respect to dehydration below 5–10% RH, although their dehydration products differ. While dihydrate D dehydrates to form I, dihydrate B converts concomitantly to forms II and III, and dihydrate E structure collapses to produce amorphous OZPN.

At high relative humidity (up to at least 95% RH), dihydrates B and D appeared by GVS to be quite stable in the solid state. This required independent confirmation by, for example, PXRD analysis, since polymorph conversions do not involve weight changes observable by gravimetric methods. The moisture sorption isotherm of dihydrate E, on the other hand, showed a reversible uptake of 0.5 molar equivalents of water between 70 and 80% RH which occurs with rapid conversion to the ‘higher hydrate’, see Fig. 6 ▸(*d*). The plateau in water composition at the high end of the RH range shows that the higher hydrate is in fact a stoichiometric hydrate retaining 2.5 molar equivalents of water (2.5 hydrate), which is in excellent agreement with the crystal structure determination (Reutzel-Edens *et al.*, 2003 ▸ *a*). The reversion of the 2.5 hydrate to dihydrate E below 75% RH helps to explain why it was so difficult to maintain the hydration state of this form while harvesting freshly grown crystals [Fig. 5 ▸(*a*)].

Given the impact that hydrate formation can have on solid-form development, it is important to know if there are other hydrates to be found. In fact, we have compelling evidence of a crystalline monohydrate, produced at room temperature by partial dehydration of dihydrate B at 0% RH (section S1 of the supporting information). Unfortunately, as a desolvation product, we could neither grow single crystals for SCXRD analysis nor produce this material in pure enough form for structure solution from PXRD. A (higher) hydrate structure (CSD refcode: AQOMEY01) in the space group *P*2_1_/*c* was also reportedly grown from an ethyl acetate–water mixture (Wawrzycka-Gorczyca *et al.*, 2007 ▸). This disordered structure is closely related, based on a root mean square deviation of 20 molecule (RMSD_20_) overlays, to isostructural dihydrate B (AQOMAU03, RMSD_20_ = 0.260 Å), the acetic acid solvate (QEPWUF, RMSD_20_ = 0.346 Å) and the ethanol solvate (MICHIR, RMSD_20_ = 0.349 Å). As such, the designation of this structure as a hydrate is tenuous at best, given that the solvent was not unambiguously identified, and hydrolysis of ethyl acetate in water produces acetic acid and ethanol, both of which could just as easily be incorporated in the disordered structure (Fig. S5). Here, SCXRD analysis alone is not sufficient for form identification; an orthogonal chemical characterization approach is required in such cases to identify and quantify solvent incorporation in the crystal structure.

Assessing the completeness of solid-form screening is more challenging for hydrates than for neat polymorphs, where CSP is increasingly used to complement experiments. A CSP study has yet to be conducted for OZPN hydrates of any stoichiometry in order to compare predicted low-energy structures against the hydrates that have been found to date. It should be noted that, though CSP studies of hydrates are gaining popularity, it is not yet practical to do these considerably more expensive calculations on all of the most common hydrate stoichiometries, let alone the unusual ones or non-stochiometric compositions.

## 5. Structures, stability, serendipity   

Crystalline forms are used to isolate, store and deliver drug molecules for the vast majority of solid oral dosage forms. Generally, once the solid form of the active pharmaceutical ingredient is selected, a scalable crystallization process is developed to produce the preferred form in high yield, controlling particle size and shape, and rejecting any unwanted impurities. The design of a robust crystallization process requires the same thorough understanding of the solid-form landscape employed in form selection, as crystallization processes can only be designed for and around forms that are known. This thorough understanding is achieved by not only identifying neat polymorphs, hydrates and any solvates from process-relevant solvents, but also establishing their stability relationships and interconversion pathways. For OZPN, the final decision to develop stable form I for Zyprexa was straightforward; the challenge came earlier in sifting through the maze of putative solid forms to compile the solid-form landscape, and thereafter in navigating it to selectively crystallize form I at scale.

**Figure 7:**
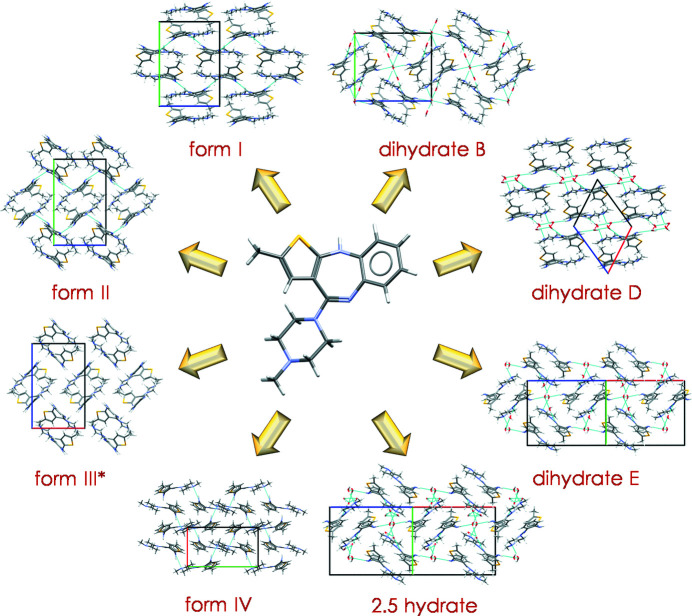
Crystal structure landscape of OZPN anhydrates and hydrates. The dispersion-bound OZPN dimer is found in all 60+ crystal forms of OZPN, except form IV.

Cursory inspection of the scientific and patent literature on OZPN shows how apparently easy it has been to lose track of its known crystal forms and the difficulty encountered in properly identifying new ones. For OZPN, it was not uncommon to produce phase mixtures or metastable forms and so a unique PXRD pattern was not necessarily the signature of a new form. SCXRD has provided invaluable confirmation of many of the OZPN crystal forms over the years, but without ssNMR spectroscopy, form III, dihydrate E and the 2.5 hydrate might still be missing. Without GVS, thermal analysis and some form of chemical characterization, isostructural disordered solvates could not be distinguished from one another. Finally, without CSP, neither would a structure model resembling form III have been generated nor the inspiration to find form IV been possible. The crystal structure landscape of the OZPN anhydrates and hydrates, which was many years in the making, is shown in Fig. 7 ▸.

OZPN, having been crystallized in 60+ forms, joins a distinguished group of highly crystallizable drug compounds, that among others, include sulfa­thia­zole (Bingham *et al.*, 2001 ▸), carbamazepine (Childs *et al.*, 2009 ▸), axitinib (Chekal *et al.*, 2009 ▸; Campeta *et al.*, 2010 ▸) and galunisertib (Bhardwaj *et al.*, 2019 ▸). It stands apart, however, for having one of the most thoroughly characterized structure landscapes, a testament to how well the compound crystallizes, the need in some cases for structure confirmation, and the general interest in exploring structure relationships underpinning form stability and transformation pathways. Remarkably, OZPN does not show the same conformational diversity across its many crystal structures as other highly polymorphic compounds, for example, ROY (Yu, 2010 ▸), flufenamic acid (López-Mejías *et al.*, 2012 ▸), aripiprazole (Zeidan *et al.*, 2016 ▸) or *R*-encenicline hydro­chloride (Kons *et al.*, 2019 ▸). With one exception (form IV), a single conformer and its enantiomer form identical dispersion-bound dimers that pack in different, energetically competitive ways. The conserved packing arrangements of OZPN in its many solvate structures (Bhardwaj *et al.*, 2013 ▸) have inspired efforts to use Random Forest modelling to predict crystal packing as a function of the solvent of crystallization (Bhardwaj *et al.*, 2018 ▸). The classification model based on 28 solvates identified the van der Waals volume, the number of covalent bonds and the polarizability of the solvent as key contributors to direct the 3D crystal packing type and led to the discovery of a novel 1-propanol solvate via targeted crystallization.

Understanding form stability, especially the risks of hydrate formation during processing and storage, was critical to the development of the OZPN drug product. Although exceptions are known (Reutzel-Edens *et al.*, 2003*b* ▸; Stephenson *et al.*, 2012 ▸), conversions to hydrates usually lead to decreased aqueous solubility and poorer dissolution. Thus, with hydrate formation all but expected to compromise the OZPN drug product, given the already low aqueous solubility of form I, it was imperative that conditions be identified to preserve form I in the drug product throughout its shelf life. Paisana *et al.* have reported long-term stability studies at 25°C, contrasting the hydration pathways and kinetics of forms I and II/III in the solid state as a function of RH. Forms II/III are converted to dihydrate B at 93% RH and to dihydrates B and E (possibly the 2.5 hydrate) at 75% RH, whereas form I was shown to gradually, over the course of 180 days, absorb moisture with partial conversion to dihydrate D at 93% RH (Paisana *et al.*, 2016 ▸). The distinct hydration pathways of forms I and II/III in the solid state were reasoned by their structural similarity to different hydrates on the crystal structure landscape (Fig. 7 ▸). OZPN dimers are aligned end-to-end in form I and its hydration product, dihydrate D, whereas dimers in the crystal structures of forms II/III and its hydration products, dihydrates B and E (or the 2.5 hydrate), have a herringbone arrangement.

**Figure 8:**
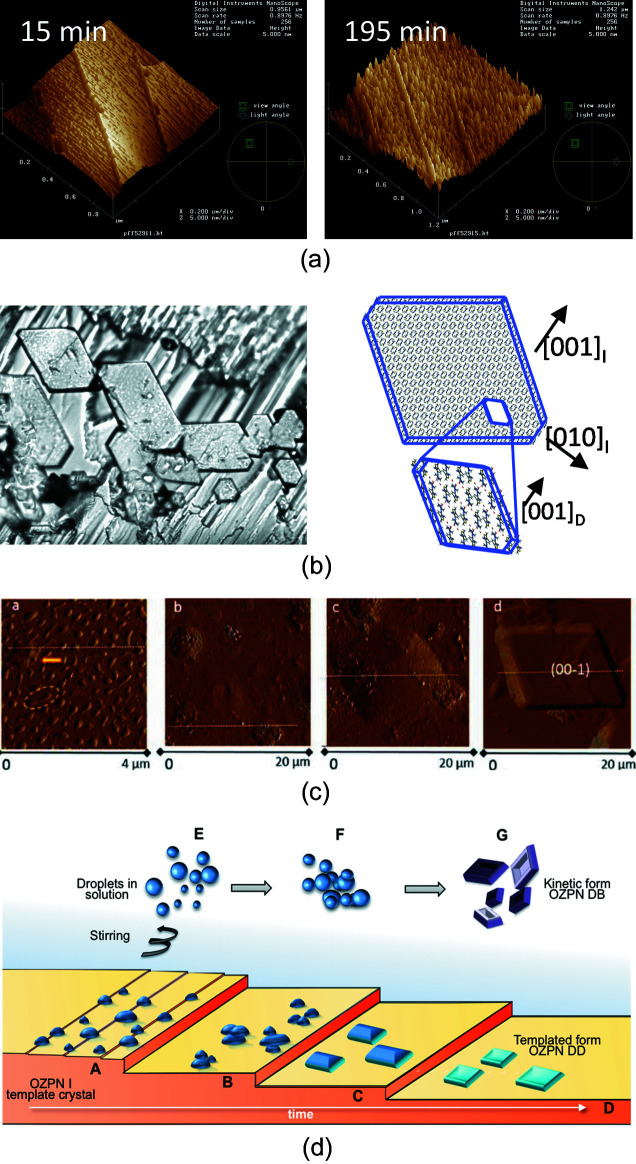
(*a*) AFM image of the OZPN form I (100) face after 15 and 195 min at 50% RH, (*b*) epitaxial growth of dihydrate D crystals on the (100) face of form I in water, (*c*) AFM image showing incipient evolution of surface features on the (100) face of form I in water, and (*d*) mechanistic view of the two-step nucleation of OZPN dihydrates D and B on and off the (100) surface of form I, respectively. Adapted from the work by Warzecha, Guo *et al.* (2017 ▸).

It is generally assumed that phase transformations at high RH are the same, only slower, than those in water slurries. However, for OZPN form I, different hydration pathways were observed in stirred water suspensions (indirect conversion to dihydrate D via dihydrate B) and in the solid state (direct conversion to dihydrate D). To contrast the hydration events in solution with those mediated by water vapour, an atomic force microscopy (AFM) study was conducted to examine the effects of water activity on OZPN hydrate formation. In view of the rather unremarkable GVS isotherm of form I [Fig. 6 ▸(*a*)], the observations using AFM at controlled RH did not disappoint. As shown in Fig. 8 ▸(*a*), the largest (100) face of a freshly cleaved form I single crystal grown from ethyl acetate is characterized by layers, each with a step height of 10 Å, the interlayer spacing along the crystallographic *a* axis. To our surprise, within ∼3 h at 50% RH, the smooth layers of OZPN in form I gave way to highly textured terraces covered with hillocks. Similar observations were made at 35% RH (Warzecha, Guo *et al.*, 2017 ▸), well below the long-term stability condition established for form I.

Unable to identify the thin overlayer of hillocks, we suspended the form I crystal in a quiescent water solution and within two days, well formed, oriented crystallites, shown by Raman microspectroscopy to be dihydrate D, could be seen under a light microscope attached to the (100) face [Fig. 8 ▸(*b*)]. The initial hydration to either dihydrate B or dihydrate D appeared to depend on the stirring condition, prompting further investigation into the impact of local mixing on dihydrate formation. Here, single crystals of form I were immersed in two saturated (with respect to form I) water solutions, one with stirring, the other without. After 48 h, hydrate crystals could be seen on the largest (100) face of both form I crystals: misaligned and loosely attached dihydrate B crystals were grown with stirring, while oriented and strongly adhered dihydrate D crystals grew from the quiescent solution (Warzecha, Guo *et al.*, 2017 ▸).

Form I, in still water and presumably also at high RH, appeared to template the crystallization of dihydrate D. Using *in situ* AFM to follow the events leading to surface hydration, the hillocks concentrated at the steps of the (001) face were confirmed to be dense solute-rich nanodroplets (liquid–liquid phase separation). Without stirring, the water-saturated OZPN nanodroplets coalesced on the surface of form I, followed by an apparent ordering and eventual crystallization of dihydrate D [Fig. 8 ▸(*c*)]. With stirring, the droplets were effectively swept from the surface of form I, allowing dihydrate B to crystallize on its own as the kinetic form. These results, along with the subsequent observation of dense mesoscopic (∼35 nm radius) OZPN-rich clusters in water and ethanol–water mixtures (Warzecha, Safari *et al.*, 2017 ▸), showed that dihydrate formation involves a two-step nucleation mechanism, see Fig. 8 ▸(*d*). Recently, the self-assembly of OZPN that leads to non-classical crystal growth and strongly biases OZPN to crystallize in dimer-based forms was demonstrated for the first time. In this work, crystals of the OZPN ethanol–water mixed solvate (WEXQEW) were shown to grow dimer by dimer in solutions comprised of predominantly monomers, driven by the preferential adsorption and overwhelming accumulation of dimers on the surface where they are most accessible to the high-energy kink sites of the growing crystal (Warzecha *et al.*, 2020 ▸).

The rich crystal chemistry of OZPN has been revealed over the better part of three decades, from the earliest days of form discovery during the development of Zyprexa to the recent study of molecular self-assembly during crystallization using AFM and molecular dynamics simulations. We now understand why it is virtually impossible to isolate forms II and III as pure polycrystalline phases, why intervention was required to break the dispersion-bound dimer in order to crystallize form IV, and why single crystals of dihydrate E could not be grown. Although we cannot be certain that new crystal forms will not one day appear, we do have mounting evidence, with the help of crystal structure prediction, that the most stable neat polymorph has already been found. At a fundamental level, OZPN has taught us that electrostatic interactions (*e.g.* hydrogen bonding) are not necessarily the most important pairwise intermolecular interactions in crystal structures. This means that crystal engineering based on Etter’s rule of hierarchical hydrogen bonding (Etter, 1990 ▸) and polymorph risk assessments based on, for example, hydrogen bonding propensity (Galek *et al.*, 2007 ▸) must be placed in context with the competition of highly favourable dispersion interactions (Section S2 of the supporting information). Finally, OZPN has shown us that the contribution of even low levels of dimers and clusters to crystal nucleation and growth in solution is not insignificant (Reutzel-Edens, 2020 ▸). Establishing the growth unit in crystallization is, for obvious reasons, critical for the high-fidelity modelling and simulation of form-dependent properties, such as crystal shape (Sun *et al.*, 2018 ▸). There is little doubt that, moving forward, it will also be essential for predicting conditions to target forms in crystallization processes.

Be it through systematic study, curiosity or serendipity, our understanding of the solid-state chemistry of OZPN has helped to ensure the safety and efficacy of commercial OZPN products that rely on exquisite control over physical properties, in some cases, showing the need for improvement. With many analytical, modelling and simulation tools that have been developed and tested on OZPN over the years being increasingly applied to small molecules in development pipelines, this remarkable drug continues to shape the way solid forms are developed and commercialized to this day.

## 6. Conclusions   

We set out to highlight, through the lens of OZPN, the importance of crystals and crystal chemistry in pharmaceutical development. While we have attempted to assemble many independent investigations of OZPN published over many years, along with a few previously undisclosed results, into a cohesive story, it is clear that the collective understanding of the solid-state chemistry of OZPN will only continue to evolve. In time, this report will join the ranks of previous studies in painting what is a partial picture of the amazingly complex crystal chemistry of this important drug molecule. Nonetheless, it is our hope that we have brought enough clarity to what is known about the crystal forms of OZPN to finish this first book. Now, let the sequel begin.

## 7. Related literature   

The following references are cited in the supporting information: Antzutkin (1999 ▸); Fung *et al.* (2000 ▸); Gavezzotti (1994 ▸); Gavezzotti & Filippini (1994 ▸); Macrae *et al.* (2020 ▸); Metz *et al.* (1994 ▸); Wood *et al.* (2013 ▸).

## Supplementary Material

Detailed experimental methods and supplemental characterization data. DOI: 10.1107/S2052252520012683/yc5024sup1.pdf

## Figures and Tables

**Table 1:** Crystallographic data for neat polymorphs and hydrates of OZPN

	Form I	Form II	Form III†	Form IV‡	Dihydrate B	Dihydrate D	Dihydrate E	2.5 Hydrate
CSD refcode	UNOGIN03	UNOGIN04	–	UNOGIN05	AQOMAU03	AQOMAU	AQOMAU02	AQOMEY2
Crystal system	Monoclinic	Monoclinic	Orthorhombic	Monoclinic	Monoclinic	Monoclinic	Monoclinic	Monoclinic
Space group	*P*2_1_/*c*	*P*2_1_/*c*	*Pbca*	*P*2_1_/*n*	*P*2_1_/*c*	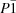	*C*2/*c*	*C*2/*c*
*a* (Å)	10.3411 (13)	9.8544 (14)	10.3454	8.6555 (2)	9.846 (2)	9.927 (5)	24.5195	24.940 (6)
*b* (Å)	14.521 (2)	16.314 (2)	19.5267	15.4441 (10)	12.672 (3)	10.095 (5)	12.3495	12.156 (3)
*c* (Å)	10.5314 (14)	9.9754 (12)	16.528	12.5558 (9)	14.384 (3)	10.514 (6)	15.2179	14.867 (3)
α (°)	90	90	90	90	90	84.710 (10)	90	90
β (°)	100.291 (4)	98.304 (8)	90	95.284 (4)	92.724 (9)	62.665 (8)	125.824	124.928 (6)
γ (°)	90	90	90	90	90	71.183 (8)	90	90
Volume (Å^3^)	1555.9 (4)	1586.9 (4)	3338.85	1671.28	1792.7 (7)	884.1 (8)	3736.3	3695.2 (14)
*Z*	4	4	8	4	4	2	8	8
*D*_calc_ (g cm^−3^)	1.334	1.308	1.243	1.242	1.291	1.309	1.239	1.285
*T* (K)	123	123	298	443	123	128	298	123
Reference	Bhardwaj *et al.* (2013 ▸)	Bhardwaj *et al.* (2013 ▸)	Bhardwaj *et al.* (2013 ▸)	Askin *et al.* (2019 ▸)	Bhardwaj *et al.* (2013 ▸)	Reutzel-Edens *et al.* (2003 ▸ *a*)	Reutzel-Edens *et al.* (2003 ▸ *a*)	Bhardwaj *et al.* (2013 ▸)

† Best form III (form III*) CSP structure from the two-phase Pawley-type refinement of match (A162) to laboratory PXRD data.

‡ Structure obtained from the Rietveld fit of the CSP structure model to synchrotron PXRD data.
