# Supplementary material for: Crystal forms in pharmaceutical applications: olanzapine, a gift to crystal chemistry that keeps on giving
Source: IUCrJ. 2020 Oct 3;7(Pt 6):955–64. doi: 10.1107/S2052252520012683 (PMC7642794; doi:10.1107/S2052252520012683)
Supplement: Supplementary file 1 [file m-07-00955-sup1.pdf]

IUCrJ

Volume 7 (2020)

Supporting information for article:

Crystal forms in pharmaceutical applications: olanzapine, a gift to crystal chemistry that keeps on giving

Susan M. Reutzel-Edens and Rajni M. Bhardwaj

S1. Solid-state characterization of OZPN polymorphs and hydrates

The dehydration of OZPN dihydrates B, D and/or E was studied as a function of temperature by differential scanning calorimetry-thermogravimetric analysis (DSC-TGA). Whereas a single desolvation event was observed for both dihydrates D and E, a bimodal weight loss is discernible for dihydrate B when heated at 10 °C/min, Figure S1. The feasibility of removing one molar equivalent of water from dihydrate B was demonstrated by moisture sorption analysis and in low temperature vacuum drying experiments. The best OZPN monohydrate material was isolated by storing dihydrate B in a 0% RH chamber for ~24 hours, then in a 22% RH chamber overnight.

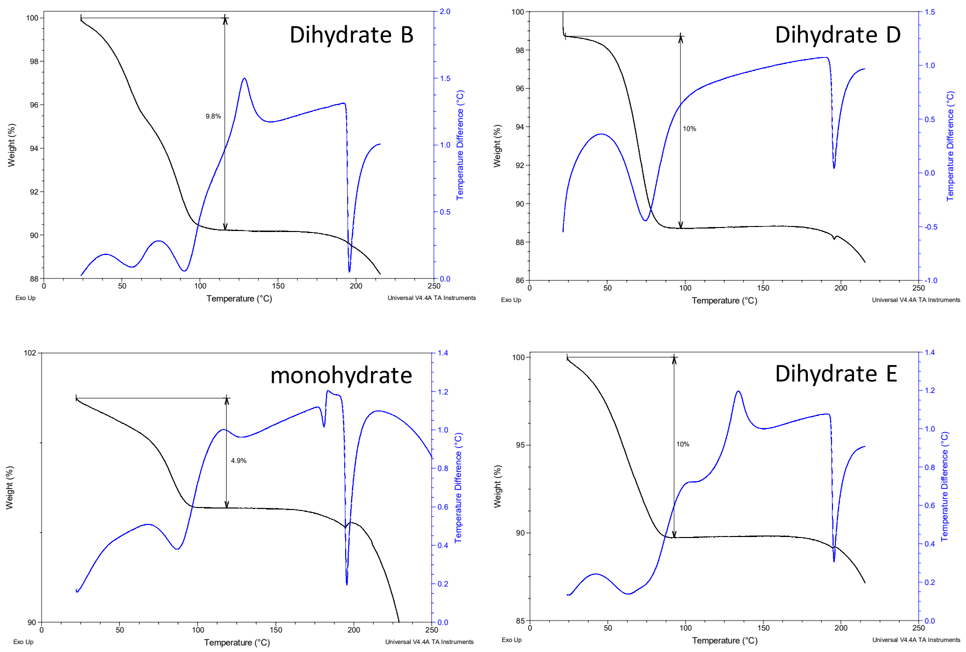

Figure S1. DSC-TGA traces of olanzapine monohydrate and dihydrates B, D and E measured at 10 °C/min.

The powder X-ray diffraction (PXRD) pattern of OZPN monohydrate, along with reference patterns for forms I-III and dihydrates B, D, and E, are shown in Figure S2. In being nearly isostructural to forms II and III, the monohydrate PXRD pattern resembles that of the metastable neat polymorphs. By contrast, the ssNMR spectrum of this hydrate is readily differentiated from the neat polymorphs and hydrates of OZPN, Figure S3. As a superior technique for identifying OZPN forms, solid-state ^{13}C NMR spectroscopy clearly showed even the best monohydrate material to be contaminated with forms II and III.

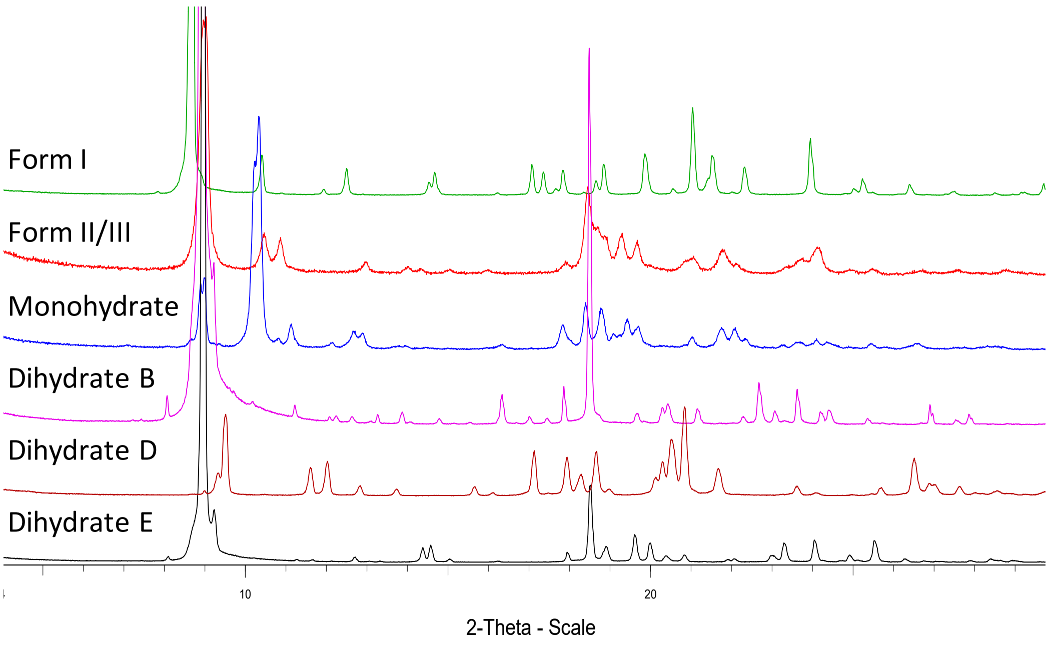

Figure S2. PXRD patterns of OZPN forms.

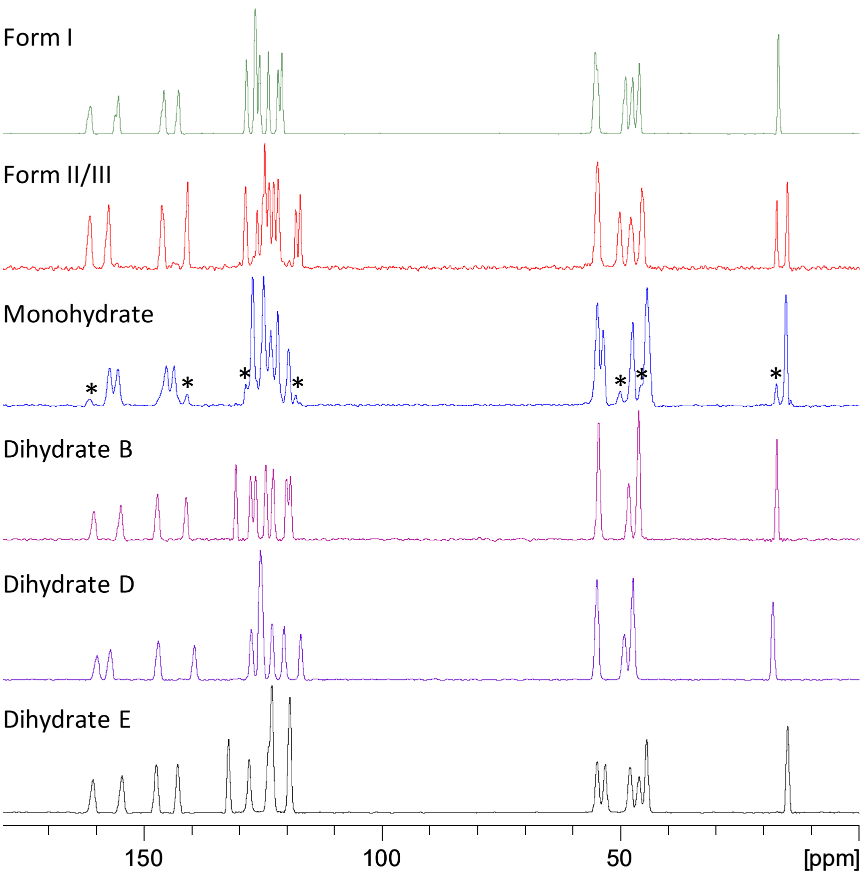

Figure S3. Solid-state ^{13}C NMR spectra of OZPN crystal forms. Form II and III impurity peaks in the monohydrate spectrum are denoted with asterisks (*).

A wetcake of freshly crystallized 2.5 hydrate, prepared according to published procedures (Reutzel-Edens *et al.*, 2003), was characterized by PXRD and ssNMR spectroscopy for comparison to its partial dehydration product, dihydrate E. The isostructural 2.5 hydrate and dihydrate E show small differences by PXRD, Figure S4. With the incorporation of an additional 0.5 waters of crystallization, the symmetry of the 2.5 hydrate is lower than that of dihydrate E. This is clearly seen by ssNMR spectroscopy, with all of the ^{13}C peaks of OZPN doubled in the spectrum of the 2.5 hydrate.

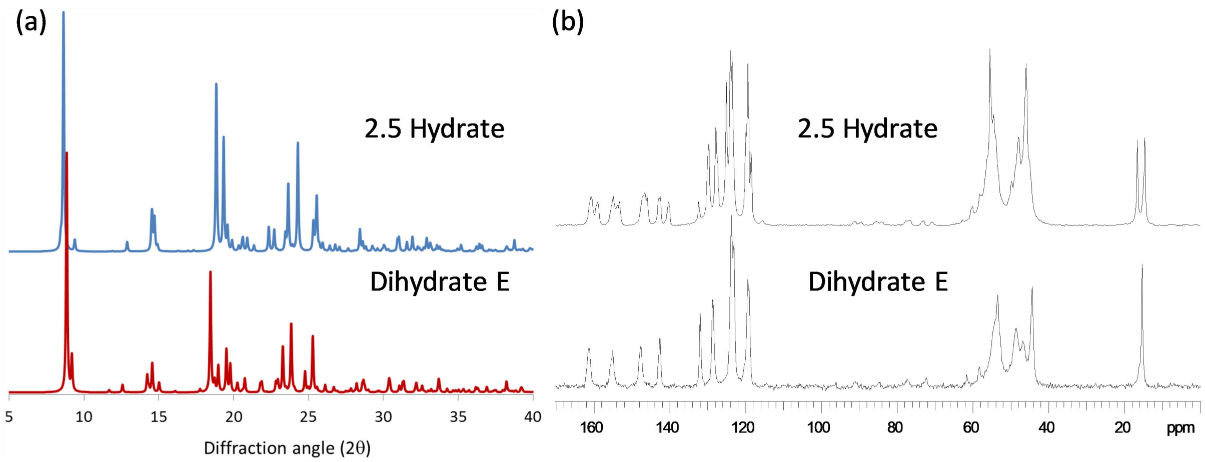

Figure S4. (a) Simulated PXRD patterns and (b) experimental ^{13}C ssNMR spectra of 2.5 Hydrate and dihydrate E.

A putative hydrate structure (CSD refcode: AQOMEY01) was reportedly grown from ethyl acetate-water (Wawrzycka-Gorczyca *et al.*, 2007), Figure S5a. This disordered $P2_1/c$ structure is closely related, based on root mean square deviation of 20 molecule overlays ($RMSD_{20}$), to dihydrate B (AQOMAU03, $RMSD_{20}$ = 0.260 Å), the acetic acid solvate (QEPWUF, $RMSD_{20}$ = 0.346 Å) and the ethanol solvate (MICHIR, $RMSD_{20}$ = 0.349 Å), Figure S5b. The solvent was not unambiguously identified from the AQOMEY01 hydrate crystal structure. Given that the hydrolysis of ethyl acetate in water produces acetic acid and ethanol and each of these solvents could account for the electron density in the disordered structure (Figure S5c), the crystallization of a hydrate could not be confirmed.

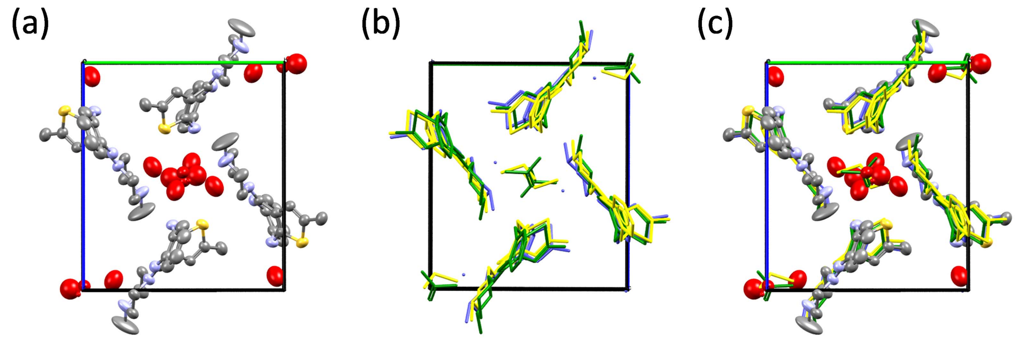

Figure S5. (a) Crystal structure of a putative OZPN hydrate (AQOMEY01, OZPN colored by element, solvent(s) shown in red), (b) crystal structure overlay of acetic acid solvate (QEPWUF, green), ethanol solvate (MICHIR, yellow), and dihydrate B (AQOMAU03, blue), and (c) crystal structure overlay of all four OZPN crystal structures.

S2. Solid form informatics

The hydrogen bonding interaction preferences of OZPN in the experimental crystal structures of forms I-IV were examined in relation to the near neighbor hydrogen bonding groups through visualization of full interaction maps (Wood *et al.*, 2013). Full interaction maps were calculated for OZPN form I and IV using NH donor and carbonyl acceptor probes to highlight where donor and acceptor partners are expected to be found based on similar structures in the CSD. Only two acceptor N atoms (diazepine N1 and piperazine N4) compete for the sole H-bond donor (N3H) of OZPN, as evidenced by just three hydrogen bonding "hotspots" for each molecule in the dimer, Figure S6. The NH donor and both N acceptors in the dispersion bound OZPN dimer are outward facing, where they are available for hydrogen bonding to other dimers or the solvent(s) of crystallization (in the hydrates and solvates). In forms I-III, dimers are linked into two-dimensional layers by NH···N hydrogen bonds between N1 and NH3; acceptor N4 is not used. In the catemeric form IV, an additional, small hotspot is seen near the piperazine N2, although this potential acceptor is not used. Instead, OZPN monomers are linked into chains by NH···N hydrogen bonds between N4 and NH3. The hydrogen bonding geometries in forms I-III conform to the expected donor and acceptor positions derived from similar molecules, while the hydrogen bonding interaction to N4 in form IV is slightly distorted (the N and NH lie at the outskirts of the hotspot).

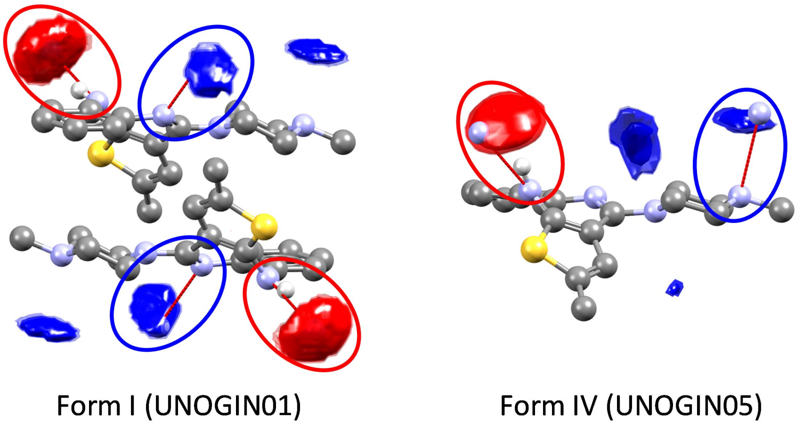

Figure S6. Full interaction maps of OZPN in forms I and IV. Hotspots for carbonyl acceptors are shown in red; hotspots for NH donors are shown in blue. Different donor-acceptor pairings are seen in the dimer-based polymorphs (I-III) than in catemeric form IV. The hydrogen bond geometries in forms I-III are well represented by similar structures in the CSD; the hydrogen bonding partners in form IV lie just outside the hotspots, suggesting a slightly unusual geometry for this intermolecular interaction. CH hydrogens are omitted for clarity.

As a complement to PIXEL calculations, UNI intermolecular potentials (Gavezzotti, 1994, Gavezzotti & Filippini, 1994) were calculated for OZPN forms I and IV as implemented within Mercury. This method, which uses empirical pair-potential parameters, provides approximate intermolecular interaction energies. As shown in Figure S7, the dispersion bound dimer is the most important pairwise interaction in the crystal structure of form I (UNOGIN01), in agreement with PIXEL calculations (Bhardwaj *et al.*, 2013). The dimer is also stronger than any pairwise interaction in the crystal structure of form IV.

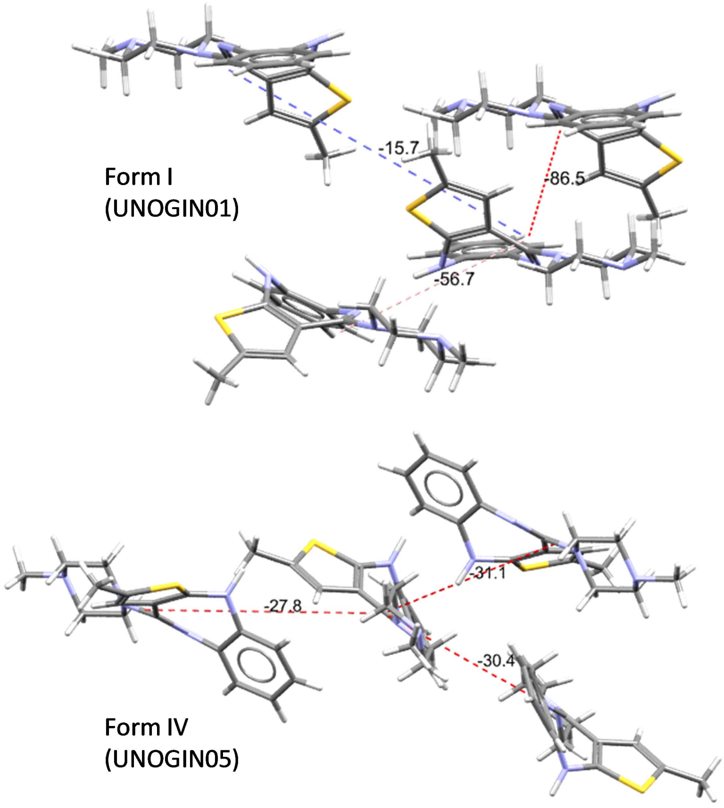

Figure S7. UNI intermolecular potentials, showing that the dispersion bound dimer is the most important pairwise interaction in the crystal structure of form I (UNOGIN01) and stronger than any pairwise interaction in catemeric form IV (UNIGON05). Intermolecular interaction energies are given in kJ/mol.

S3. Methods

PXRD patterns were collected on a Bruker D4 Endeaver X-ray powder diffractometer, equipped with a CuKα source (λ=1.54056 Å) and a Vantec detector, and operating at 40 kV and 50 mA, with 0.06 mm divergence and detector slits. Each sample was scanned from 4 to 40º in 0.009º 2θ steps at a rate of 0.2 seconds per step.

Cross polarization/magic angle spinning NMR spectra were obtained on a Bruker Avance III 400 wide-bore NMR spectrometer operating at ^{1}H and ^{13}C frequencies of 400.131 and 100.622 MHz, respectively, and using either a Bruker 4 mm double-resonance or a 4 mm triple-resonance probe. The MAS rate was set to 10 kHz using a Bruker MAS-II controller; spinning speeds were maintained within 2 Hz of the set point. SPINAL64 (Fung *et al.*, 2000) decoupling at a proton nutation frequency of 100 kHz was used for heteronuclear decoupling. Spinning sidebands were eliminated by a five-pulse total sideband suppression (TOSS) sequence (Antzutkin, 1999). The CP contact time for transferring magnetization from protons to carbons was set to 1.5 ms and a linear power ramp from was used on the ^{1}H channel to enhance CP efficiency (Metz *et al.*, 1994). The acquisition time was

set to 34 ms and spectra were acquired over a spectral width of 30 kHz with 616 transients and a recycle delay of 5 s. The sample temperature was regulated to 297 ± 1 K in order to minimize frictional heating caused by sample spinning. The ^{13}C chemical shifts were externally referenced (± 0.05 ppm) to the proton-decoupled ^{13}C peak of neat (liquid) tetramethylsilane via the high-field resonance of adamantane (δ = 29.5 ppm).

Moisture sorption analysis was performed at 25 °C using a VTI flow moisture balance (Model SGA-100). The following experimental conditions were used: sample size 25-30 mg, 30-95-1-95% RH programmed adsorption/desorption cycle, 5% RH step increment.

Differential thermal/thermogravimetric analyses were carried out on a TA simultaneous DSC-TGA unit (Model SDT Q600). Samples were heated in open aluminum pans from about 20 to 250 ºC at 10 ºC/min with a nitrogen purge of 100 mL/min. The temperature was calibrated with indium. The weight calibration was performed with a manufacturer-supplied standard.

References

Antzutkin, O. N. (1999). *Progress in Nuclear Magnetic Resonance Spectroscopy* **35**, 203-266.

Bhardwaj, R. M., Price, L. S., Price, S. L., Reutzel-Edens, S. M., Miller, G. J., Oswald, I. D. H., Johnston, B. F. & Florence, A. J. (2013). *Crystal Growth & Design* **13**, 1602-1617.

Fung, B. M., Khitrin, A. K. & Ermolaev, K. (2000). *Journal of Magnetic Resonance* **142**, 97-101.

Galek, P. T. A., Fábián, L., Motherwell, W. D. S., Allen, F. H. & Feeder, N. (2007). *Acta Crystallographica Section B* **63**, 768-782.

Gavezzotti, A. (1994). *Accounts of Chemical Research* **27**, 309-314.

Gavezzotti, A. & Filippini, G. (1994). *The Journal of Physical Chemistry* **98**, 4831-4837.

Macrae, C. F., Sovago, I., Cottrell, S. J., Galek, P. T. A., McCabe, P., Pidcock, E., Platings, M., Shields, G. P., Stevens, J. S., Towler, M. & Wood, P. A. (2020). *Journal of Applied Crystallography* **53**, 226-235.

Metz, G., Wu, X. L. & Smith, S. O. (1994). *Journal of Magnetic Resonance, Series A* **110**, 219-227.

Reutzel-Edens, S. M., Bush, J. K., Magee, P. A., Stephenson, G. A. & Byrn, S. R. (2003). *Crystal Growth & Design* **3**, 897-907.

Wawrzycka-Gorczyca, I., Borowski, P., Osypiuk-Tomasik, J., Mazur, L. & Koziol, A. E. (2007). *Journal of molecular structure* **830**, 188-197.

Wood, P. A., Olsson, T. S. G., Cole, J. C., Cottrell, S. J., Feeder, N., Galek, P. T. A., Groom, C. R. & Pidcock, E. (2013). *CrystEngComm* **15**, 65-72.
